# A practical approach for incorporating dependence among fields in probabilistic record linkage

**DOI:** 10.1186/1472-6947-13-97

**Published:** 2013-08-30

**Authors:** Joanne K Daggy, Huiping Xu, Siu L Hui, Roland E Gamache, Shaun J Grannis

**Affiliations:** 1 Department of Biostatistics, Indiana University School of Medicine, Indianapolis, IN, USA; 2 Regenstrief Institute and Indiana University School of Medicine, Indianapolis, IN, USA

## Abstract

**Background:**

Methods for linking real-world healthcare data often use a latent class model, where the latent, or unknown, class is the true match status of candidate record-pairs. This commonly used model assumes that agreement patterns among multiple fields within a latent class are independent. When this assumption is violated, various approaches, including the most commonly proposed loglinear models, have been suggested to account for conditional dependence.

**Methods:**

We present a step-by-step guide to identify important dependencies between fields through a correlation residual plot and demonstrate how they can be incorporated into loglinear models for record linkage. This method is applied to healthcare data from the patient registry for a large county health department.

**Results:**

Our method could be readily implemented using standard software (with code supplied) to produce an overall better model fit as measured by BIC and deviance. Finding the most parsimonious model is known to reduce bias in parameter estimates.

**Conclusions:**

This novel approach identifies and accommodates conditional dependence in the context of record linkage. The conditional dependence model is recommended for routine use due to its flexibility for incorporating conditional dependence and easy implementation using existing software.

## Background

Health information exchanges (HIE’s), with highly heterogeneous data, are becoming increasingly important sources of integrated clinical data supporting many healthcare tasks and health-related research. HIE data are captured from different independent databases with different patient identifiers, and best practices for implementing and operating HIE’s are needed. Specifically with respect to data integration and patient matching, in its formal recommendations to the Director of the Office of the National Coordinator for Health Information Technology (HIT) in 2011, the HIT Policy Committee recognized the need to develop and disseminate best practices for patient matching [1] because best practices for matching data in HIE’s are lacking.

Many methodologies have been proposed to identify records in two or more databases that are related to the same entity. Deterministic approaches are based on ad-hoc rules, which classify a pair of records as matches if the two records satisfy certain conditions. Although straightforward to implement, deterministic approaches are often too conservative with unacceptably high false negative (missed-match) rates, especially when data are noisy [2]. This may lead to suboptimal care since physicians lack the information necessary to make informed medical decisions.

Distance-based methods that can handle numerical or categorical fields, as described in [3], are another method to link records. These methods have been shown to perform similarly to probabilistic methods for both numeric [4] and categorical data [5] but require one to establish appropriate distance measures for each variable under consideration. They are not investigated further here as they are not commonly used in practice and have not yet been investigated thoroughly in the HIE setting although they may be of interest in future work [6].

Another alternative to deterministic linkage methods are probabilistic methods. A common probabilistic record linkage method was proposed by Fellegi and Sunter in 1969 [7]. This model is a latent class model, where the latent, or unknown, class represents the *true match status* of the record pair. For this model, each field contained in both data sources is compared as a record pair and a binary variable is created which is a 1 if the two fields agree and 0 otherwise; thus a binary vector is created for each record pair. The Fellegi-Sunter (F-S) model assumes that the agreement patterns of the fields are independent conditional on the true match status.

This conditional independence assumption is often violated in real-world record linkage scenarios [8]. When conditional independence does not hold, estimates of model parameters can be substantially biased [9]. This bias can lead to inaccurate record linkage outcomes as described previously [2,8]. Therefore, finding the most parsimonious model that accounts for the conditional dependence will provide the most accurate classification of record pairs.

Various methods have been proposed to address the lack of conditional independence in latent class models for record linkage. For example, Tromp et al. incorporated conditional dependence between two fields by combining them into one field with four nominal levels of agreement [2]. This strategy can be cumbersome if conditional dependence exists between more than two fields since the number of nominal categories increases when combining agreement patterns for multiple fields. Schürle proposed an alternate approach to incorporating conditional dependence in the traditional F-S model framework by working directly with the joint distribution of the observed agreement pattern given the true match status. However, this model involves heavy parameterization that leads to significant overfitting of the model [10]. For example, when seven fields are used for record matching, this model involves 255 parameters, while the data could estimate at most 127 parameters. Due to the extreme complexity of the model, the proper choice of starting values is critical for parameter estimation. This greatly limits the usefulness of the approach due to the computational effort required to examine multiple starting values.

Latent class models with conditional independence can be equivalently formulated using a loglinear framework [11,12]. Using this formulation, the conditional independence assumption can be readily relaxed to account for conditional dependence among fields by including interactions among fields within the match class or the nonmatch class or both [13]. Such loglinear approaches incorporating interactions within latent classes have been used in many applications, notably in diagnostic testing [14,15].

Similarly, loglinear models have been applied to record linkage applications. Using survey data whose record pairs had known match status, Thibaudeau identified fields with conditional dependence using a loglinear model with selected interactions [8]. Winkler estimated a loglinear model using three-way interactions, acknowledging that identifying the correct set of interactions is difficult when a large number of fields are involved [16]. Loglinear models with certain interaction terms have also been applied in record linkage by Larsen and his colleagues [17,18]. There has been no research on effectively identifying appropriate interactions in record linkage until the stepwise model building strategy for identifying interactions recently proposed by Zhu et al. [19]. However, this approach can only identify models with all interactions of the same order.

Many previous record linkage studies focused largely on maximum likelihood (ML) estimation, where the parameter estimates of the loglinear model were obtained using an Expectation-Maximization (EM) algorithm. For situations such as the latent class model where incomplete data (unobserved classes) are involved, the EM algorithm is a powerful tool to estimate model parameters [20]. However, as noted by Winkler (1995), the EM algorithm takes substantially longer to reach convergence when conditional dependence is incorporated in the loglinear model because the M-step does not have a closed-form solution [21]. Alternatively, estimating the loglinear latent class model can be conveniently implemented using routines in existing software, such as SAS® PROC NLMIXED (Cary, NC), thus providing a pragmatic approach to incorporating conditional dependence more efficiently.

Even though loglinear models have been proposed by multiple authors for handling conditional dependence in HIE, implementation of such models requires customized programs and the process for choosing pairwise interactions in these models has not been specified. We therefore describe and evaluate a method for identifying conditional dependence among fields, which are subsequently incorporated as interactions in a loglinear model fitted using standard software. To illustrate the methodology, we use an application linking a client list of a county health department to itself for de-duplication. The step-by-step method described is supplemented by sample code which can be readily modified for linking any two data sets using standard statistical software.

## Methods

We first describe a loglinear formulation of the extended F-S model with conditional dependence. Let *M* be the true match status of a pair of records (*M=* 1 for true match and *M=* 0 for true non-match). For each record pair with *K* fields, an agreement vector is observed

$$Y=\left\{y_{1},y_{2},\ldots ,y_{K}\right\},$$

where y_i_ = 1 if the *i*^*th*^ field agrees and 0 otherwise. The match prevalence is defined as the proportion of vector patterns belonging to the true match record class and is π = *P*(*M =* 1). The parameters of the classical F-S model include

$$\Theta =\left\{m_{1},m_{2},\ldots ,m_{K},u_{1},u_{2},\ldots ,u_{K},\pi \right\}$$

where the *m*-probabilities are the probability of field agreement given the record pair is a true match, and the *u*-probabilities are the probabilities of field agreement given the record is a true non-match.

To more effectively accommodate conditional independence, the traditional F-S model can be reparameterized using a loglinear formulation, where the mean number of record pairs with agreement pattern *Y* and match status *M* is given as follows:

(1) $$\text{log}\;m\left(Y,M\right)=\lambda +\lambda_{M}M+\sum_{k=1}^{K}\lambda_{k}y_{k}+\sum_{k=1}^{K}\lambda_{\mathrm{Mk}}My_{k}.$$

With *K* fields, there are *D =* 2^*K*^ possible different agreement patterns. Let *f*_*d*_ represent the frequency count for the agreement pattern *Y*_*d*_ (*d = 1,2,…,D*). Then the log-likelihood is given by

$$l\left(\Theta |Y\right)=\sum_{{}^{d=1}}^{D}f_{d}\text{log}\left(P\left(Y_{d}\right)\right),$$

where the marginal probability of observing the agreement pattern *Y*_*d*_ is

(2) $$P\left(Y_{d}\right)=\frac{m\left(Y_{d},M_{d}=1\right)+m\left(Y_{d},M_{d}=0\right)}{\;\Sigma \begin{array}{c} {}_{D}\\ {}^{d=1} \end{array}\left\{m\left(Y_{d},M_{d}=1\right)+m\left(Y_{d},M_{d}=0\right)\right\}}.$$

The match score for a specific agreement pattern *Y*_*d*_ is defined as

$${\text{log}}_{2}\left\{\frac{P\left(Y_{d}|M_{d}=1\right)}{P\left(Y_{d}|M_{d}=0\right)}\right\}.$$

The loglinear formulation has been shown to be equivalent to the F-S classical probabilistic formulation of the conditional independence latent class model [12] through the following relationships:

$$\begin{array}{c} \pi =\frac{\Sigma \begin{array}{c} {}_{D}\\ {}^{d=1} \end{array}m\left(Y_{d},M_{d}=1\right)}{\Sigma \begin{array}{c} {}_{D}\\ {}^{d=1} \end{array}m\left(Y_{d},M_{d}=1\right)+\Sigma \begin{array}{c} {}_{D}\\ {}^{d=1} \end{array}m\left(Y_{d},M_{d}=0\right)},\\ m_{k}=\frac{\exp\left(\lambda_{k}+\lambda_{\mathrm{Mk}}\right)}{1+\exp\left(\lambda_{k}+\lambda_{\mathrm{Mk}}\right)},\\ u_{k}=\frac{\exp\left(\lambda_{k}\right)}{1+\exp\left(\lambda_{k}\right)}. \end{array}$$

To incorporate conditional dependence in the loglinear model setting, we add the appropriate interaction terms to the model. For example, if there is dependence between fields *j* and *l* within each latent class, the model then includes two additional terms:

(3) $$\begin{array}{l} \log\;m\left(Y,M\right)=\lambda +\lambda_{M}M+\sum_{k=1}^{K}\lambda_{k}y_{k}+\sum_{k=1}^{K}\lambda_{\mathrm{Mk}}My_{k}\\ \;+\lambda_{\mathrm{jl}}y_{j}y_{l}+\lambda_{\mathrm{Mjl}}My_{j}y_{l}. \end{array}$$

The above loglinear model with interaction terms is easy to fit in standard statistical software such as SAS (example code is provided in Additional file 1). The goodness of fit of a model is measured by both the deviance G^2^ and the Bayesian Information Criterion (BIC). We use deviance to compare nested models. A model with lower deviance provides a better fit to the data and hence will be preferred. For models that are not nested within each other, BIC is the most commonly used criterion for latent class modeling as it takes into account the sample size [22]. The model with a lower BIC is preferred.

In what follows, we describe a series of steps to fit a loglinear model with appropriate interactions. Specifically, we follow a six-step procedure by identifying the pairwise dependencies between fields using the correlation residual plot proposed by Qu, Tan, and Kutner [23]. We then incorporate the correlations into the model and re-examine the fit of the new model. We iterate between these steps as follows:

### Step 1

Fit a loglinear model with no interactions using the observed agreement vectors. This is simply the F-S model formulated as a loglinear model, which provides initial parameter estimates for the next model. Obtain deviance and BIC of this conditional independence model. See Additional files 1, 2 and 3 for SAS code with example.

### Step 2

Compute the observed pairwise correlation between fields *j* and *l*. The correlation between *y*_*j*_ and *y*_*l*_ is

(4) $${\mathrm{Corr}}_{\mathrm{jl}}=\frac{p_{\mathrm{jl}}-p_{j}p_{l}}{\sqrt{p_{j}\left(1-p_{j}\right)p_{l}\left(1-p_{l}\right)}},$$

where *p*_*j*_ *= P*(*y*_*j*_ *=* 1) , *p*_*l*_ *= P*(*y*_*l*_ *=* 1), and *p*_*jl*_ *= P*(*y*_*j*_ *=* 1,*y*_*l*_ *=* 1). Using the observed data, the estimates for *p*_*j*_, *p*_*l*_, and *p*_*jl*_ are given by:

$$\frac{\sum_{d=1}^{D}f_{d}y_{\mathrm{dj}}}{\sum_{d=1}^{D}f_{d}},\frac{\sum_{d=1}^{D}f_{d}y_{\mathrm{dl}}}{\sum_{d=1}^{D}f_{d}},\mathrm{and}\;\frac{\sum_{d=1}^{D}f_{d}y_{\mathrm{dj}}y_{\mathrm{dl}}}{\sum_{d=1}^{D}f_{d}},$$

respectively.

### Step 3

Substitute the parameter estimates of *λ*’s from the fitted model in *Step 1* into Equation (1) to obtain the expected number of record pairs *m(Y*_*d*_*,M*_*d*_*)* for each vector pattern *Y*_*d*_ and match status *M*_*d.*_ Calculate the expected marginal probability *P*(*Y*_*d*_) using Equation (2) and the expected cell count ${\hat{f}}_{d}=\mathrm{NP}\left(Y_{d}\right)$ for each vector pattern, where $\;N=\sum_{d=1}^{D}f_{d}$ is the total number of record pairs. Expected pairwise correlations are then estimated using (4) (same formulas in *Step 2*) based on the expected counts ${\hat{f}}_{d}$ rather than the observed counts $f_{d}$.

### Step 4

Compute the correlation residual, which is equal to the difference between the observed correlation and the expected correlation for each pair of fields. Plot the residuals across the different pairs of fields. A correlation residual which is much different from zero would imply dependence for the corresponding pair of fields.

### Step 5

Incorporate the conditional dependence between the pair of fields identified in *Step 4* as the interaction term in the loglinear model. Specifically, fit the following four models: interaction in the match class only, interaction in the nonmatch class only, interaction in both classes with different coefficients, and interaction in both classes with the same coefficients. Since the four models are not all nested, BIC is used to compare them and the model with the lowest BIC is chosen. Repeat *Steps 3 through 5* to obtain the expected number of record pairs *m*(*Y*_*d*_*,M*_*d*_) by substituting the parameter estimates of *λ*’s of the chosen model into Equation (3) with appropriate interactions instead of Equation (1) until no large correlation residuals are apparent.

### Step 6

To classify individual pairs as match, non-match or uncertain matches, we use the final model parameter estimates to calculate the match score for each agreement pattern. Record pairs are then declared as matches or non-matches based on these match scores.

Approval to perform this study was obtained from the Indiana University Institutional Review Board: approval number 1010002784 (0909–68). De-identified data for the HIE example described in the next section is provided as Additional file 3.

## Results

### Description of the HIE dataset

We applied the above steps to de-duplicate the client registry for the Marion County Health Department (MCHD). De-duplication is a class of record linkage where a data set is linked to itself to identify potential duplicate records. MCHD is a member of the Indiana Network for Patient Care, the nation’s largest and longest tenured HIE [24].

The MCHD client registry contains 779,466 patient records gathered from multiple public health service areas. Therefore this data is highly heterogeneous and the method of input may be any combination of standardized electronic entry, paper entry, or manual entry for a given field. Since the total number of all potential record pairs is extremely large (3 × 10^11^ potential pairs), the data were first blocked to minimize the search space for potential matches. The MCHD client registry was blocked on last name and first name, thus only record pairs agreeing on these two fields are contained in the analysis. This reduced the number of potential record pairs to 618,213. The remaining fields in this dataset include day, month, and year of birth, social security number, telephone number, zip code, and gender. Level of missing across the different fields varies from as low as 0% for day, month, and year of birth to as high as 95% for specific identifiers such as SSN. Missing values were coded as disagreements. We then applied the six-step process described above to all pairs from this block.

### Application to HIE dataset

**Figure 1:**
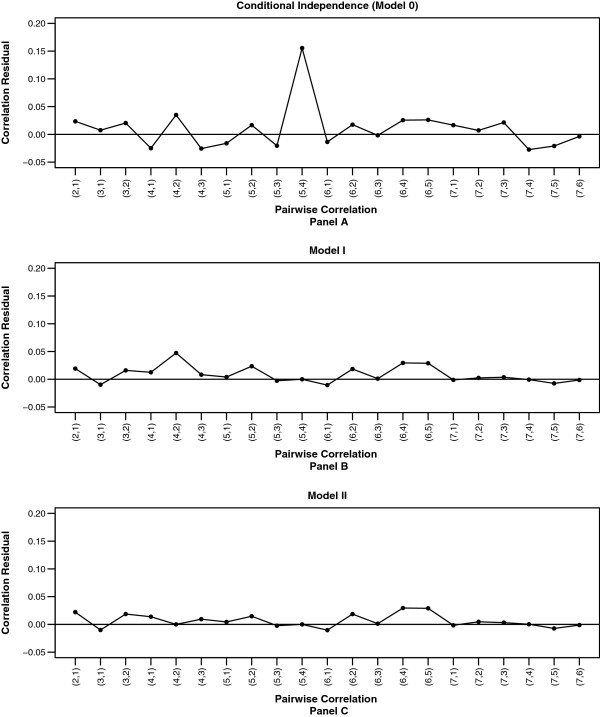
**Correlation residual plots for last name/first name block.** Last name/first name block: pairwise correlation residuals for Model 0 **(**Panel **A)**, Model I **(**Panel **B)**, and Model II **(**Panel **C)**.

As described in the previous section, we first fit the conditional independence model (*Step 1*). With 7 fields, this model contains 15 parameters (Model 0). Parameter estimates are provided in Table 1. The overall match prevalence was estimated to be 3.7% under this model. The *m*-probabilities ranged from a low of .025 (SSN) to a high of 0.716 (month of birth), indicating that only 2.5% of the matched record pairs agreed on SSN while 71.6% agreed on month of birth. The estimated *u*-probabilities were small as expected, except for sex, which was estimated to be 0.661, indicating that 66.1% of non-matching record pairs agreed on sex. Also, the *u*-probability for SSN was nearly zero, indicating that very few non-matching record pairs agree on SSN. The deviance of this model was G^2^ = 8852.9. Assuming independence, the observed and expected pairwise correlations were calculated (*Steps 2, 3*) and the differences were displayed in the correlation residual plot (*Step 4*), as shown in Figure 1 (Panel A).

**Table 1:** Loglinear model results for last name/first name block

		**Model 0**		**Model I**		**Model II**	
		**(Conditional independence)**					
**Field**	**Parameter**	**Estimate**	**Std Error**	**Estimate**	**Std Error**	**Estimate**	**Std Error**
	***π***	**0.037**	**0.0004**	**0.035**	**0.0004**	**0.035**	**0.0004**
Year of birth	*m*_*1*_	0.581	0.0047	0.615	0.0048	0.615	0.0048
SSN	*m*_*2*_	0.025	0.0011	0.027	0.0011	0.026	0.0011
Day of birth	*m*_*3*_	0.572	0.0046	0.608	0.0048	0.608	0.0048
Telephone	*m*_*4*_	0.173	0.0029	0.141	0.0026	0.140	0.0026
Zip code	*m*_*5*_	0.409	0.0041	0.363	0.0040	0.362	0.0040
Sex	*m*_*6*_	0.710	0.0037	0.695	0.0038	0.694	0.0038
Month of birth	*m*_*7*_	0.716	0.0044	0.768	0.0044	0.769	0.0045
Year of birth	*u*_*1*_	0.026	0.0002	0.026	0.0002	0.026	0.0002
SSN	*u*_*2*_	6E-06	8E-06	1E-05	9.00E-06	5E-05	1E-05
Day of birth	*u*_*3*_	0.032	0.0003	0.031	0.0003	0.031	0.0003
Telephone	*u*_*4*_	5E-04	0.0001	0.002	0.0001	0.002	0.0001
Zip code	*u*_*5*_	0.037	0.0003	0.039	0.0003	0.039	0.0003
Sex	*u*_*6*_	0.661	0.0006	0.661	0.0006	0.661	0.0006
Month of birth	*u*_*7*_	0.082	0.0004	0.081	0.0004	0.081	0.0004
G^2^		8852.9		2974.26		2881.45	

Loglinear model results for MCHD data blocked on last name and first name (Number of record pairs = 618,213). All parameters are statistically significant (p < .001) for all three models, except for u_2_ which is not significant for conditional independence model (p = .468) or Loglinear Model I (p = .143).

The seven fields in this particular dataset yield 21 pairwise correlations. The difference between the 21 observed and expected pairwise correlations from this model ranged from −0.027 to 0.155. The majority of the correlation residuals from the conditional independence model fluctuate between −0.03 and 0.05. However, the correlation residual between the fields telephone number and zip code is much larger than the others (almost 5-fold difference), indicating a violation of the conditional independence assumption for this pair of variables.

To accommodate the conditional dependence between telephone number and zip code, we followed *Step 5* and compared the fit of four models, specifically, models with interaction in the match class only, interaction in the non-match class only, interaction in both classes with different coefficients, and interaction in both classes with equal coefficients. The model with interactions between telephone number and zip code in both classes with different coefficients yielded the lowest BIC and hence was selected (Model I).

The parameter estimates of the match prevalence and *m*- and *u*-probabilities for Model I are also shown in Table 1. Model I provided a better fit to the data compared to the conditional independence model, which is indicated by its much lower deviance = 2974.3. The estimated match prevalence was 3.5%, slightly lower than the estimate under the conditional independence model. The estimated *u*-probabilities were quite similar under both models. The difference was seen in the estimated *m*-probabilities, with an increase in the estimates corresponding to year, month, day of birth and SSN and a decrease in estimates corresponding to telephone, zip code and sex compared to the conditional independence model.

After calculating the expected correlations under Model I (repeat *Step 3*), Figure 1 (Panel B) shows the correlation residuals for pairs of fields (repeat *Step 4*). The correlation residual for telephone number and zip code is no longer present since this pairwise dependence has been accounted for by the model. The magnitude of all correlation residuals were under 0.05. Our experience suggests that an absolute value greater than 0.05 for the correlation residual is a reasonable approximate guideline for identifying conditional pairwise field dependence.

Although the correlation residual plot did not reveal substantial deviation of the conditional dependence between other pairs of fields, the correlation residual between SSN and telephone number (0.047) was more than twice of the magnitude of the remaining residuals. To examine whether it is appropriate to consider conditional dependence between this pair of fields, we repeated the *Step 5* and compared the fit of four additional models. These four models again include models with interaction between SSN and telephone number in the match class only, interaction in the non-match class only, interaction in both classes with different coefficients, and interaction in both classes with equal coefficients. Based on the BIC, the model with interactions in both classes with different coefficients was selected (Model II).

Model II provided a better fit to the data compared to Model I with a smaller deviance and BIC, as well as smaller correlation residuals (Figure 1 Panel C). However, the improvement of the model fit was marginal. This is indicated by its deviance G^2^ = 2881.45, which was only slightly less than the deviance of Model I. As a result, parameter estimates under Model II did not differ much from the less complex Model I. As this is consistent with our general guideline regarding correlations above .05, we chose Model I as our final model.

Patient records were then classified as match or non-match based on the estimated match prevalence from these three models. The conditional independence model classified 1,152 record pairs as matches. Model I classified 1,082 matches and Model II results were almost identical to Model I with 1,081 matches. Thus not accounting for the conditional dependence yielded the largest number of declared duplicates; likely producing falsely-merged records resulting in lost patient records. Since it was not the purpose of our study to assess the accuracy of the different models, we did not manually ascertain the true match status of the records. We refer the readers to the literature [2,7] for examples where accounting for the conditional dependence may improve the performance of the matching algorithm.

## Discussion

For many record linkage applications, the assumption of conditional independence for field agreement is often violated and ignoring the conditional dependence may lead to a suboptimal record matching accuracy. To optimize matching accuracy, it is important to examine whether conditional dependence exists and to incorporate such dependence in the model in a proper way.

In this paper, we presented a step-by-step procedure to identify and incorporate conditional dependence among fields using a loglinear latent class model. This stepwise method can be implemented using standard statistical software. In contrast to previous studies where loglinear latent class models were estimated using the iterative EM algorithm, we proposed estimating parameters using the readily available SAS procedure NLMIXED. Our step-by-step process was applied to the de-duplication of the MCHD client registry. The results indicated that conditional dependence can be readily identified using a graphical approach and the model with appropriate conditional dependence provided a much better model fit than the conditional independence model.

Although a stepwise variable-selection strategy was previously proposed by Zhu et al. [19] that accounted for the conditional dependence using interaction terms in loglinear models, the model building process proposed in this paper is different in several important ways. First, the quasi-Newton approach implemented in SAS NLMIXED procedure used in this paper is more efficient than the EM algorithm. Second, the final model obtained by the Zhu et al. approach must include all interactions of the same order, while our approach can identify specific two-way interactions. When a large number of fields are involved in record linkage, examining all interactions of the same order will introduce a large number of additional parameters. Model estimation can become quite complex with many local maxima. In addition, important conditional dependencies may not be detected using Zhu et al.’s approach when only a few interaction effects exist.

In addition to loglinear models, latent class models with conditional dependence have been extensively studied and widely used in other domains. For example, in diagnostic testing, latent class models with random effects [23,25], probit latent class models [26], and finite mixture models [27] have all been used to evaluate the accuracy of diagnostic tests when no gold standard is available to evaluate the true disease status. Further investigation is needed to examine how the other conditional dependence models compare to loglinear models and to determine the impact of incorporating conditional dependence in record linkage.

A potential limitation of our approach is that it is more labor intensive because it requires understanding how to fit loglinear models. Additionally, the loglinear model framework requires parameterization that is not as readily understood by practitioners. The approach is iterative thus is also more computationally intensive. However, these challenges are mitigated by providing example code as additional files.

## Conclusions

We have proposed a novel and practical approach to identify and incorporate conditional dependence in record linkage. Compared to the commonly used F-S model, the conditional dependence model provides substantially better fit to the data when conditional dependence exists. Given that some fields commonly used for linking health records often have correlated agreement patterns, we recommend the routine use of our proposed methods to avoid model misfit. Our approach can be easily followed using the step-by-step instructions and the sample code provided.

## Competing interests

The authors declare that they have no competing interests.

## Authors’ contributions

JKD drafted the manuscript, prepared tables and figures as well as supplemental material. HX performed analysis, was involved in drafting manuscript and created SAS macro. SLH reformatted and also wrote portions of the manuscript. REG provided knowledge on record linkage. This work was supported by SJG’s grant and SJG provided additional knowledge on record linkage as well as provided the data. All authors read and approved the final manuscript.

## Pre-publication history

The pre-publication history for this paper can be accessed here:

http://www.biomedcentral.com/1472-6947/13/97/prepub

## Supplementary Material

Additional file 1 **MCHD Loglinear Models.sas.** SAS program that uses the loglinear approach described in this paper to fit the MCHD dataset. Requires SAS® software available from SAS Institute Inc., Cary, NC, USA. Click here for file

Additional file 2 **corr macro.sas.** SAS macro to compute the correlation residual. Requires SAS® software available from SAS Institute Inc., Cary, NC, USA. Click here for file

Additional file 3 **MCHD data.csv.** MCHD data supplied in standard csv format. Click here for file
